# The Severity of Direct Antiglobulin Test Negative ABO Hemolytic Disease of Newborn: A Retrospective Analysis at a Tertiary Children’s Hospital

**DOI:** 10.1007/s12288-023-01689-4

**Published:** 2023-08-26

**Authors:** Huimin Ma, Zhe Sheng, Jin Xu

**Affiliations:** Department of Clinical Laboratory, Children’s Hospital of Fudan University, National Children’s Medical Center, Shanghai, 201102 China

**Keywords:** ABO-HDN, Elution test, Hyperbilirubinemia, MRI

## Abstract

This study aimed to evaluate the severity of ABO hemolytic disease of newborn (ABO-HDN) with negative direct antiglobulin test (DAT), which was identified by elution test. We retrospectively reviewed the clinical records of all neonates admitted with the diagnosis of neonatal hyperbilirubinemia requiring phototherapy or exchange transfusion. Neonates were divided into four groups according to their immunohematology test results. Then their essential laboratory results, magnetic resonance image (MRI), brainstem auditory evoked potential (BAEP) findings, and rate of exchange transfusion were compared between different groups. We found that neonates in ABO-HDN with negative DAT group developed jaundice faster and anaemia more severely than those in the non-HDN group. Although they might get less severe anaemia than neonates in ABO-HDN with positive DAT group and the Rh-HDN group, neonates in ABO HDN with negative DAT group might develop jaundice as quickly as the latter two groups. As to MRI and BAEP findings, there were no significant differences among the four groups. The rate of exchange transfusion in ABO-HDN with negative DAT group was higher than that in the non-HDN group but lower than that in ABO-HDN with positive DAT group, though without statistical significance. It suggested that in the presence of clinical suspicion of ABO-HDN with negative DAT result, the elution test should be added to rule out or confirm the diagnosis to help prevent the morbidity from hyperbilirubinemia.

## Introduction

Immunohematology tests, mainly direct antiglobulin test (DAT), serum antibody test, and elution test, are of limited value in predicting hemolytic disease of newborn (HDN) caused by ABO incompatibility (ABO-HDN). Most newborns with positive immunohematology test results don’t necessarily progress to hyperbilirubinemia [1], but some may also go rapidly to severe anaemia, clinically significant hyperbilirubinemia, requiring continuous phototherapy or exchange transfusion [2, 3]. Newborns with hemolytic disease have less tolerance for bilirubin encephalopathy (BE), which might be irreversible [4]. So for newborns who have progressed to hyperbilirubinemia, it is essential to identify those with immunohematology aetiology. In recent years, many ABO-HDN cases have been reported in which newborns with severe hyperbilirubinemia were found to have negative DATs but positive elution tests [5–7]. Nevertheless, the elution test is not performed universally due to its long turnaround time, so there is little study concerning the severity of elution-confirmed ABO-HDN compared with other kinds of HDN or nonspecific hyperbilirubinemia.

Our hospital is one of the few pediatric hospitals in the largest city in eastern China which admits neonates with hyperbilirubinemia and can perform exchange transfusions. This retrospective study aimed to evaluate the severity of anaemia and hyperbilirubinemia in DAT-negative ABO-HDN newborns identified by elution tests and its damage to the central nervous system.

## Methods

### Patients

This retrospective study covered one year, from January to December of 2021. Inclusion criteria included newborns with estimated gestational age > 35 weeks, admitted with the diagnosis of neonatal hyperbilirubinemia requiring phototherapy or exchange transfusion, and postnatal age of no more than 28 days. Neonates with other risk factors for neonatal hyperbilirubinemia, such as low birth weight, neonatal sepsis, cephalic hematoma, hypothyroidism, asphyxia, or other conditions causing hemolysis such as glucose-6-phosphate dehydrogenase (G6PD) deficiency were excluded. All patients were born outside birthing facilities. The clinical records of all neonates were reviewed for demographics, laboratory parameters, and postnatal management. The ethics committee of the hospital approved this retrospective study.

### Laboratory Methods of Detection and Diagnosis of HDN in Infants

Screening for HDN was routinely performed on neonates admitted with a diagnosis of hyperbilirubinemia. ABO and rhesus (D) blood type, irregular antibody screening and three qualified HDN screening tests (DAT, serum antibody test and elution test) were determined on neonates' EDTA whole blood using the gel method according to manufacturer’s instructions (Diagnostic Grifols, S.A., Spain).

The heat elution was conducted according to laboratory protocols. Firstly the neonate’s red blood cells were washed three times in cold saline, then the packed cells were added to the same volume of saline. The suspension was subjected to 56 °C in a water bath for 10 min, then centrifuged at 3400 rpm for 2 min. The supernatant saline eluate was quickly siphoned off to a new tube at the end of the centrifugation. The eluate obtained above was then subjected to a microcolumn gel card (Diagnostic Grifols, S.A., Spain) to react with adult A/B/O red blood cells. When antibodies were present, agglutination of corresponding A or B red blood cells could be shown.

The acid elution method followed the manufacturer’s instructions (Shanghai Hemo-Pharmaceutical & Biological Co., Ltd, Shanghai, China). The neonate’s washed packed cells were added to reagent A, gently shaking for 15 s. The suspension was centrifuged at 3400 rpm for 1 min; then, the supernatant was siphoned off to a new tube. The reagent B was added to the supernatant drop by drop until the blue supernatant eluate was obtained. The eluate was then subjected to a microcolumn gel card to react with antibody screen cells panel (Shanghai Hemo-Pharmaceutical & Biological Co., Ltd, Shanghai, China) or antibody identification cells panel (Sanquin Reagents B.V., The Netherlands).

If the antigen was present on the red blood cells and the same antibody was detected in the serum or the eluate, it was judged to be a positive elution test or serum antibody test.

For maternal–fetal ABO incompatibility, a positive elution test (heat-elution method [8]) would confirm the laboratory diagnosis of ABO-HDN, regardless of the DAT and serum antibody test results. For other maternal–fetal blood type incompatibilities, a positive irregular antibody screen and a positive direct antiglobulin test would be followed by an elution test (acid-elution method). Then antibody identification would be made on both serum and eluate. Detection of the same antibody in both serum and eluate confirmed the diagnosis of HDN caused by that antibody.

### Other Tests, Examinations and, Treatment Options for the Neonates

Together with evidence of hemolysis, a laboratory-confirmed diagnosis of HDN could be considered a high-risk factor for lowering the threshold for phototherapy or exchange transfusion according to unit protocols based on the 2004 American Academy of Pediatrics (AAP) hyperbilirubinemia treatment guidelines or for the administration of intravenous immunoglobulin therapy. For ABO-HDN, exchange transfusions were performed using the combination of group O-packed cells and group AB plasma [9]. Other hematological tests included total serum bilirubin (TSB), whole blood hemoglobulin (HB), hematocrit, reticulocyte count, thyroid function and GP6D tests. In addition, sepsis-related tests such as blood cultures could be performed as options based on clinical symptoms.

Cranial magnetic resonance imaging (MRI) and brainstem auditory evoked potentials (BAEP) might be performed to identify signs of acute bilirubin encephalopathy with the informed consent of the neonate's parents. Enhanced T1W1 signal in the symmetric globus pallidus was considered abnormal MRI, and listening impairment was supposed to be abnormal BAEP.

### Data Analysis and Statistical Methods

Neonates were divided into four groups according to the immunohematology test results—non-HDN group, ABO-HDN with negative DAT group, ABO-HDN with positive DAT group and Rh-HDN group. The mean value of peak total serum bilirubin (peak TSB), minimum hemoglobin (minimum HB) during their stay in the hospital, the proportion of neonates with anaemia, MRI findings, BAEP findings and exchange transfusion rates were compared between different groups.

Statistical analysis of the data was performed using SPSS 25.0 statistical software (2017, IBM Co., NY, USA). Categorical variables were expressed as frequencies (percentages), and numerical variables were expressed as mean ± standard deviation (SD) or median (interquartile range). Categorical variables were compared by chi-square test or Fisher's exact method, and numerical variables were analysed by one way-ANOVA, Krustal-Wallis H-test, or Spearman's correlation analysis. *P* < 0.05 was considered to be of statistical significance.

## Results

### Basic Information and Test/Exam Results of the Neonates

From January to December 2021, 697 newborns were admitted to the neonatal unit diagnosed with neonatal hyperbilirubinemia and met the inclusion criteria. The demographic characteristics are shown in Table 1. None of these neonates progressed to acute bilirubin encephalopathy (ABE) or died during their stay in the hospital.

**Table 1:** Demographic characteristics of enrolled neonates (N = 697)

Characteristics	Values
Sex no. (%)	
Male	392 (56.1%)
Female	305 (43.9%)
Group no. (%)	
Non-HDN	527 (75.6%)
ABO-HDN-n DAT	143 (20.5%)
ABO-HDN-p DAT	13 (1.9%)
Rh-HDN	14 (2.0%)
Admission age median (IQR) (day)	5 (3, 7)
Body weight at birth (mean ± SD) (g)	3325.64 ± 370.16
peak TSB (mean ± SD) (umol/L)	318.26 ± 70.96
valley HB (mean ± SD) (g/L)	156.55 ± 28.26

*Non-HDN* without HDN, *ABO-HDN-n DAT* ABO-HDN with negative DAT, *ABO-HDN-p DAT* ABO-HDN with positive DAT

Values are expressed as number (%), means ± standard deviation (SD), and median with interquartile range (IQR)

According to the immunohematology test results, 527 (75.6%) neonates were divided into the non-HDN group. Of the 156 (22.4%) ABO-HDN neonates, 143 (91.67%) were confirmed ABO-HDN with negative DAT, while only 13 (8.33%) were ABO-HDN with positive DAT. All neonates with positive DAT had positive elution and serum antibody tests. Of the 143 neonates with negative DAT, the elution tests were all positive while the serum antibody may be negative. Fourteen neonates were confirmed with Rh-HDN, with anti-D in five cases, anti-DC in one case, anti-C in two instances, anti-c in one case, anti-Ce in one case and anti-E in four cases. The latter three groups were referred to as HDN groups all together.

Cranial MRI was performed in 433 infants, of which 63 (14.55%) had abnormal MRI findings described as enhanced T1W1 signal in the symmetric globus pallidus. BEAP were performed in 87 infants, of which 34 (39.08%) had abnormal results defined as hearing impairment.

### Comparison of the Essential Characteristics Between Different Groups

The sex constitution, body weight at birth, admission age, initial reticulocyte, the minimum hematocrit, hemoglobin, the proportion of neonates with anaemia, peak total serum bilirubin, MRI and BAEP findings and exchange transfusion cases in different groups were compared, as shown in Table 2.

**Table 2:** Comparison of essential characteristics between groups

Variables	Non-HDN group	ABO-HDN-n DAT group	ABO-HDN-p DAT group	Rh-HDN group	*p* value
Sex n (%)					0.007
Male	310 (59.8%)	65 (46.1%)	4 (30.8%)	8 (57.1%)	
Female	208 (40.2%)	76 (53.9%)	9 (69.2%)	6 (42.9%)	
Body weight at birth (g) (mean ± SD)	3330.6 ± 374.5	3316.6 ± 354.3	3290.4 ± 383.2	3255.0 ± 388.8	0.877
Admission age (day) median (IQR)	5 (4, 8)	3 (2, 6)	1 (1, 4)	2.5 (0, 4.3)	< 0.001
Initial reticulocyte rate (%) median (IQR)	1.7 (1.0, 2.7)	2.70 (1.6, 5.2)	6.2 (3.1, 13.6)	5.6 (3.5, 11.4)	< 0.001
Minimum haematocrit (%) median (IQR)	47.2 (42.5, 51.4)	41.9 (3.6, 47.9)	33.9 (28.6, 37.7)	26.8 (22.1, 35.0)	< 0.001
Minimum HB (g/L) median (IQR)	165.0 (148.0, 180.0)	147.0 (125.0, 167.0)	110.0 (74.5, 111.3)	86.5 (74.5, 111.3)	< 0.001
Peak TSB (umol/L) (mean ± SD)	327.6 ± 66.5	294.8 ± 70.2	268.6 ± 67.3	288.9 ± 79.2	< 0.001
Anaemia (HB < 130 g/L) n (%)	37 (7.2%)	41 (29.1%)	10 (76.9%)	13 (92.9%)	< 0.001
MRI n (%)					0.91
Normal	264 (85.7%)	84 (84%)	11 (91.7%)	11 (84.6%)	
Abnormal	44 (14.3%)	16 (16%)	1 (8.3%)	2 (15.4%	
BAEP n (%)					> 0.99
Normal	32 (59.3%)	14 (60.9%)	3 (75.0%)	4 (66.7%)	
Abnormal	22 (40.7%)	9 (39.1%)	1 (25.0%)	2 (33.3%)	
Exchange transfusion n (%)	8 (1.52%)	6 (4.14%)	1 (7.69%)	6 (42.86%)	< 0.001

*ABO-HDN-n DAT* ABO-HDN with negative DAT, *ABO-HDN-p DAT* ABO-HDN with positive DAT, *Non-HDN* without HDN, *BAEP* brainstem auditory evoked potentials, *HB* haemoglobin, *MRI* magnetic resonance imaging, *TSB* total serum bilirubin

Values are expressed as number (%), means ± standard deviation (SD), and median with interquartile range (IQR)

There was no significant difference in birth weight between the four groups; the proportion of male infants in the ABO-HDN with negative DAT group was higher than that in the non-HDN group; there was no significant difference in sex constitution between the other groups.

There was a significant difference in the admission age between the four groups (*p* < 0.001). The admission age was older in the non-HDN group than in the other three HDN groups (*p* < 0.001). There was no significant difference in the admission age between the three HDN groups.

**Fig. 1:**
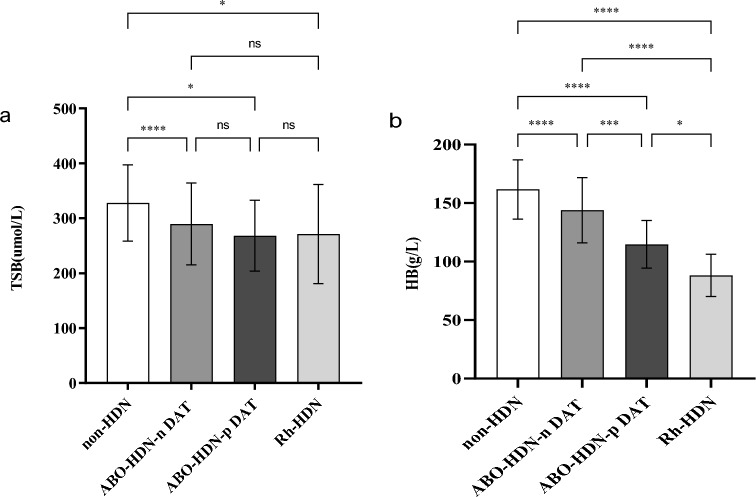
Comparison of peak TSB and minimum HB between different groups. *p < 0.05; **p < 0.01; ***p < 0.001; ****p < 0.0001; ns: not significant. **a** Comparison of peak total serum bilirubin (TSB) between different groups. **b** Comparison of minimum haemoglobin (HB) between different groups

The mean value of peak TSB levels in the non-HDN group was statistically higher than that in the HDN groups (*p* < 0.001, *p* = 0.014, *p* = 0.016, compared with ABO-HDN with negative DAT group, ABO-HDN with positive DAT group, Rh-HDN group respectively). There was no significant difference between the three HDN groups regarding the mean value of peak TSB levels. The comparison between different groups is shown in Fig. 1a.

**Fig. 2:**
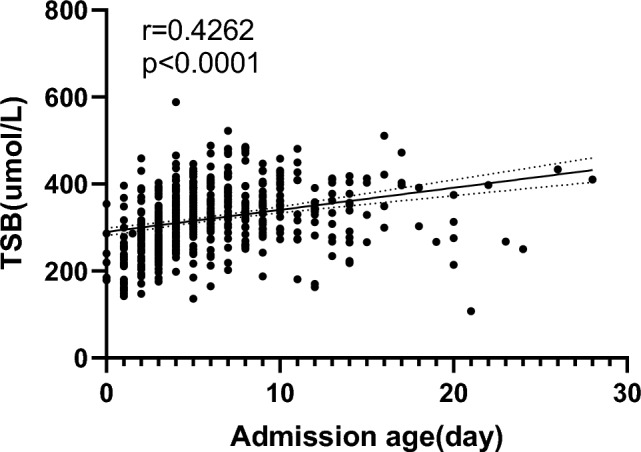
Linear correlation between total serum bilirubin (TSB) and admission age of all enrolled neonates

Spearman’s correlation analysis showed that the peak TSB levels correlated with the neonate’s admission age, with a coefficient of 0.4262 (*p* < 0.001) (Fig. 2).

The mean value of the initial reticulocyte rate in the ABO-HDN with negative DAT group was higher than that in the non-HDN group (*p* < 0.001) but lower than that in the ABO-HDN with positive DAT group and Rh-HDN group (*p* = 0.026, *p* < 0.001, respectively).

The mean value of minimum HB in the non-HDN group was higher than that in the HDN groups (*p* < 0.001); the mean value of minimum HB in the ABO-HDN with negative DAT group was higher than that in the ABO-HDN with positive DAT group and Rh-HDN group (*p* = 0.001, *p* < 0.001, compared with ABO-HDN with positive DAT group, Rh-HDN group respectively), while the mean value of minimum HB in the ABO-HDN with positive DAT group was higher than that in the Rh-HDN group (*p* = 0.047). The comparison between different groups is shown in Fig. 1b.

Anaemia was diagnosed by a venous hemoglobin < 130 g/L [10]. The proportion of neonates with anaemia in the ABO-HDN with negative DAT group was lower than that in the non-HDN group and higher than that in the ABO-HDN with positive DAT group and Rh-HDN group (*p* < 0.001).

These data indicated that hemolysis progressed more severely in the ABO-HDN with negative DAT group than in the non-HDN group but less severely than in the ABO-HDN with positive DAT group and Rh-HDN group.

### Analysis of Magnetic Resonance Imaging (MRI) and Brainstem Auditory Evoked Potential (BAEP) Findings

There were no significant differences in MRI or BAEP findings between the groups, as shown in Table 2.

Based on this, we further divided infants into two groups according to their MRI and BAEP findings to analyse the relation between the mean value of peak TSB levels and MRI or BAEP findings. We found no significant difference in the mean value of peak TSB levels between the normal and abnormal MRI groups (*p* = 0.918) and between the normal and abnormal BAEP groups (*p* = 0.247).

### The Cases of Exchange Transfusion Therapy

A total of 21 neonates were administered with exchange transfusion. Those included eight in the non-HDN group (1.52%), six in ABO-HDN with negative DAT group (4.14%), one in ABO-HDN with positive DAT group (7.7%) and six in the Rh-HDN group (42.86%). Fisher's exact test showed that the rate of exchange transfusion was higher in the Rh-HDN group than that in other groups (*p* < 0.001). There wasn’t a significant difference in the rate of exchange transfusion between other groups. Some essential characteristics of these neonates are shown in Table 3.

**Table 3:** Some essential characteristics of the neonates administered with exchange transfusion

Case	Sex	Admission age (day)	Birth Weight (g)	Group	MRI	BAEP	Peak TSB (umol/L)	Minimum HB (g/L)	ET (times)
1	Female	4	3880	Non-HDN	Normal	–	588.6	128	1
2	Female	7	4080	Non-HDN	Normal	–	481	155	1
3	Male	8	3920	Non-HDN	Normal	–	483.9	143	1
4	Male	11	2650	Non-HDN	Normal	Abnormal	449.9	134	1
5	Male	13	3150	Non-HDN	Normal	Abnormal	412.8	131	1
6	Female	16	3230	Non-HDN	Normal	Normal	511.1	113	1
7	Male	22	3610	Non-HDN	Normal	Abnormal	438.3	116	1
8	Female	3	2965	Non-HDN	Normal	–	392.1	135	1
9	Female	1	3170	ABO-HDN-n DAT	Abnormal	–	396.3	80	1
10	Female	1	3070	ABO-HDN-n DAT	Abnormal	Abnormal	278.3	109	1
11	Male	1	3080	ABO-HDN-n DAT	–	–	368.6	87	1
12	Female	2	2500	ABO-HDN-n DAT	Normal	Abnormal	299.5	80	1
13	Male	2	3500	ABO-HDN-n DAT	Normal	–	430	92	1
14	Male	3	3000	ABO-HDN-n DAT	Normal	Normal	459.3	121	1
15	Female	1	3530	ABO-HDN-p DAT	Normal	–	229.8	110	1
16	Male	0	3430	RH-HDN	Normal	–	185.1	115	1
17	Female	0	3750	RH-HDN	Abnormal	–	354	86	1
18	Female	2	3250	RH-HDN	Normal	Normal	369.9	77	2
19	Female	3	2260	RH-HDN	Normal	Abnormal	325.4	66	1
20	Male	4	3220	RH-HDN	Normal	–	395.4	63	1
21	Female	4	2630	RH-HDN	Normal	Normal	369.4	121	1

*ABO-HDN-n DAT* ABO-HDN with negative DAT, *ABO-HDN-p DAT* ABO-HDN with positive DAT, *BAEP* brainstem auditory evoked potential, – not done, *MRI* magnetic resonance imaging, *Non-HDN* without HDN

Six neonates (case 9 to case 14 as shown in Table 3) were administered with exchange transfusion in ABO-HDN with negative DAT group. All six neonates had negative DAT, positive serum antibody, and positive elution tests and recovered successfully.

Compared with neonates in the non-HDN group, the six neonates in ABO-HDN with negative DAT group who received exchange transfusion had younger admission age (1.7 ± 0.8 day vs. 10.5 ± 6.4 day, *p* = 0.006) and lower minimum hemoglobin (94.8 ± 16.7 mg/L vs. 131.9 ± 13.6 mg/L, *p* = 0.001).

The ninth to twelfth neonates were found to be yellow-stained in birthing hospitals within the 24th hour after birth and had relatively higher transcutaneous bilirubin. Then they were transferred to the neonate unit of our hospital soon with an initial TSB of 233.2 umol/L, 273 umol/L, 368.6 umol/L and 274.8 umol/L respectively and with an initial reticulocyte rate of 7.3%, 17.5%, 20.5% and 9.1% respectively. They were treated with intravenous immunoglobin when confirmed ABO-HDN. With the informed consent of their parents, they were administered with exchange transfusion and soon got a steady low level of total serum bilirubin.

The thirteenth and fourteenth neonates were found to have jaundice on the second day and third day, respectively. They had an initial TSB of 421.4 umol/L and 459.3 umol/L, respectively. After several hours of phototherapy, which failed to alleviate the jaundice, they were admitted to our neonate unit. They were administered with exchange transfusion soon after confirmed ABO-HDN due to exceptionally high levels of TSB, which exceeded the exchange transfusion threshold. They were administered with phototherapy after the exchange transfusion and recovered several days later without any symptoms of acute bilirubin encephalopathy.

## Discussion

ABO-HDN is a common cause of neonatal readmission, and many neonates require phototherapy or exchange therapy. From this retrospective analysis, we know that most ABO-HDN were confirmed by elution tests other than DATs. It indicates that DAT has a significantly lower sensitivity for screening ABO-HDN than the elution test. As some experts have elucidated that the number of A or B antigens on neonatal erythrocytes is lower than that on adult erythrocytes, the elution test uses more red blood cells to get the antibody bound to neonatal erythrocytes, resulting in an amplifying effect [11].

In this study, it was found that neonates with HDN, including those in ABO-HDN with negative DAT group, were admitted to the hospital earlier after birth than those without HDN. It reveals that anaemia and jaundice progressed faster in neonates with HDN, so they were detected and admitted to the hospital earlier. That was consistent with the findings of previous studies [12].

The mean value of minimum HB levels in ABO-HDN with negative DAT group was lower than that in the non-HDN group but higher than that in ABO-HDN with positive DAT group and RH-HDN group. It revealed that neonates in ABO-HDN with negative DAT group might have an increased incidence of hemolysis due to ABO incompatibility compared to those without HDN; however the clinical course was relatively benign compared to those in ABO-HDN with positive DAT group or in the Rh-HDN group.

The mean value of peak TSB levels in neonates without HDN was higher than in the HDN groups, which is somewhat puzzling and inconsistent with the findings of other studies [1, 2]. As the peak TSB levels was found to correlate with the admission age, the lower peak TSB levels in the HDN groups may be attributed to the earlier onset of jaundice and subsequently, the earlier admission to the hospital. The timely interventions of these neonates may result in the lower mean value of peak TSB levels. In addition, the neonates included in our study were all admitted with a diagnosis of neonatal hyperbilirubinemia, so the neonates in the non-HDN group were also recognised for significant jaundice and had higher bilirubin levels. In contrast, many other studies included all neonates admitted to the neonatal unit or focused on testing cord blood to predict the possibility of afterbirth hyperbilirubinemia.

Some neonates in the ABO-HDN with negative DAT group suffered hyperbilirubinemia and anaemia as severe as those in the ABO-HDN with positive DAT group and needed to be treated with exchange transfusion. The rate of exchange transfusion was higher in ABO-HDN with negative DAT group than that in the non-HDN group, though without statistical significance. Similarly, the ABO-HDN-negative DAT group has a lower rate of exchange transfusion than ABO-HDN with positive DAT group without statistical significance, either. The diagnosis of ABO-HDN with negative DAT, confirmed by an elution test, gave valuable information to physicians. It enables timely optimisation of appropriate interventions for lowering the phototherapy or exchange threshold to prevent irreversible neurological damage from hyperbilirubinemia.

Cranial MRI and BAEP examination are considered essential tools to assist in the diagnosis of acute bilirubin encephalopathy. In the present study, 61 neonates with abnormal MRI and 34 neonates with abnormal BAEP did not have any symptoms of acute bilirubin encephalopathy, indicating that abnormal MRI findings of bilateral globus pallidus or abnormal BAEP results did not necessarily reveal symptoms of acute bilirubin encephalopathy. This finding was consistent with the low specificity of cranial MRI for the diagnosis of bilirubin encephalopathy reported by Wu et al. [13]. However, Lu et al. concluded that even in the absence of symptoms of bilirubin encephalopathy, the abnormal MRI signal represented bilirubin infiltration into the brain tissue of the neonates and bilirubin brain damage had already occurred [14]. It is believed that even in the absence of acute bilirubin encephalopathy, persistent hyperbilirubinemia may cause neurodevelopmental and hearing impairment in neonates [15, 16].

The limitation of this study was that the small number of neonates in the ABO-HDN with positive DAT group and RH-HDN group may have led to insignificant differences for some variables. In addition, the severity of neonatal hyperbilirubinemia was closely related to their hour of life, which was difficult to record due to the drawbacks of retrospective studies. This study did not classify the severity of hyperbilirubinemia according to their hour of life but only compared the mean value of peak TSB, which might not accurately reflect the severity of hyperbilirubinemia in these neonates.

In conclusion, we found that neonates in ABO-HDN with negative DAT group developed anaemia and jaundice faster and more severely than neonates in the non-HDN group, which was approximately close to neonates in ABO-HDN with positive DAT group. In the presence of clinical suspicion of HDN with a negative DAT result, the elution test should be administered to rule out or confirm the diagnosis to help prevent the morbidity resulting from hyperbilirubinemia.
